# Critical Assessment of Phenotyping Cocktails for Clinical Use in an African Context †

**DOI:** 10.3390/jpm13071098

**Published:** 2023-07-05

**Authors:** Machel Leuschner, Allan Duncan Cromarty

**Affiliations:** Department of Pharmacology, Faculty of Health Sciences, University of Pretoria, Pretoria 0084, South Africa; duncan.cromarty@up.ac.za

**Keywords:** phenotyping cocktail, African genetic diversity, personalized medicine, CYP450, P-gp

## Abstract

Interethnic and interindividual variability in in vivo cytochrome P450 (CYP450)-dependent metabolism and altered drug absorption via expressed transport channels such as P-glycoprotein (P-gp) contribute to the adverse drug reactions, drug–drug interaction and therapeutic failure seen in clinical practice. A cost-effective phenotyping approach could be advantageous in providing real-time information on in vivo phenotypes to assist clinicians with individualized drug therapy, especially in resource-constrained countries such as South Africa. A number of phenotyping cocktails have been developed and the aim of this study was to critically assess the feasibility of their use in a South African context. A literature search on library databases (including AccessMedicine, BMJ, ClinicalKey, MEDLINE (Ovid), PubMed, Scopus and TOXLINE) was limited to in vivo cocktails used in the human population to phenotype phase I metabolism and/or P-gp transport. The study found that the implementation of phenotyping in clinical practice is currently limited by multiple administration routes, the varying availability of probe drugs, therapeutic doses eliciting side effects, the interaction between probe drugs and extensive sampling procedures. Analytical challenges include complicated sample workup or extraction assays and impractical analytical procedures with low detection limits, analyte sensitivity and specificity. It was concluded that a single time point, non-invasive capillary sampling, combined with a low-dose probe drug cocktail, to simultaneously quantify in vivo drug and metabolite concentrations, would enhance the feasibility and cost-effectiveness of routine phenotyping in clinical practice; however, future research is needed to establish whether the quantitative bioanalysis of drugs in a capillary whole-blood matrix correlates with that of the standard plasma/serum matrixes used as a reference in the current clinical environment.

## 1. Introduction

Interindividual variability in medicine response contributes to adverse drug reactions (ADR), drug–drug interaction (DDI) and therapeutic failure [1,2]. The African continent carries an estimated 25% of the global disease burden despite the fact that it has only 15.5% of the world’s population [3]. There are limited data available about the burden of ADR, DDI, and therapeutic failure in Sub Saharan Africa. A cross-sectional survey at four South African hospitals found that 8.4% of admissions were related to ADR and 45% of these were preventable [4].

Sixty to eighty percent of commercially available drugs today are metabolized by the CYP450 enzymes with great interindividual and interethnic variability affecting therapeutic outcomes [5]. The oral clearance of drugs through expressed permeability-glycoprotein (P-gp) transport channels (encoded by the adenosine triphosphate-binding cassette (ABC) transporter gene (ABCB1)) are also subject to pharmacokinetic variability and recent studies have shown that many drugs metabolized by the CYP450 enzymes are also ABC transporter protein substrates, indicating that both phase I metabolism and transmembrane transport form a protective barrier against foreign substances entering the body [6].

Geographical ancestry and ethnicity influence CYP allele frequencies, resulting in worldwide variability in genotypic expression and measured phenotypes, with significant differences in treatment response, risk profile and disease prevalence [4]. Samer et al. published a detailed review on the clinical impact of known CYP450 polymorphisms on drug therapy, including a summary of the consensus dosage recommendations and guidelines based on pharmacogenetic testing of CYP450 expression [5]. A main concern is the lack of published data available on the influence of genotype on Sub-Saharan African populations. The greatest diversity in the distribution of clinically relevant CYP alleles (CYP2B6*6, CYP2C8*2, CYP2D6*3, CYP2D6*17, CYP2D6*29, CYP3A5*6 and CYP3A5*7) is found in Africa [7] and was shown to be markedly different when compared to Caucasian and Asian populations. A high level of genetic and within-population diversity was found in South African Khoisan and Black populations [8,9]. This has been illustrated with commonly used drugs to treat heart disease, which are known to be less effective in individuals of African descent relative to individuals of European descent [10]. Both the non-nucleoside reverse transcriptase inhibitors and protease inhibitors are metabolized by the CYP450 enzymes, of which CYP2B6 and CYP3A4/5 have been shown to play major roles in the pharmacokinetic variability of nevirapine [11]. Most notable is the high frequency of the CYP2B6*6 allele in Sub-Saharan African populations, which could explain the high prevalence of drug-induced adverse events reported with efavirenz and nevirapine [12]. A South African study by Dodgen et al. [13] found novel CYP2C19 alleles indigenous to the South African population that contributed to a poor correlation between predicted and measured phenotypes, highlighting the importance of considering the pharmacogenetics and unique confounders present in this population. A similar finding with CYP2C9 alleles confirms the discord between predictive and measured phenotypes, where only a small number of alleles could be successfully attributed to decreased or absent enzyme activity [14]. Our current knowledge on interindividual and interethnic differences in the South African population is, however, based upon a limited number of studies, often pooling data for all African populations, inadequately contributing to diverse genetic profiles of the population [15]. Genetic polymorphisms of the adenosine triphosphate (ATP) binding cassette transporter gene (ABCB1) influence P-gp transport protein expression and ultimately drug transmembrane transport [16]. The ABCB1 SNP variants identified are published on the NCBI’s bdSNP database [17]. One SNP with extensive interethnic variability is 3435C>T with the 3435TT polymorphism resulting in lower intestinal P-gp expression and elevated plasma concentrations of digoxin on average compared to homozygous C allele carriers [18], of which the frequency of the latter genotype was found to be significantly higher in African populations compared to African American or Caucasian populations [19].

In addition to genotype, several intrinsic and extrinsic factors influence the activity of these drug-metabolizing enzymes and transmembrane transport proteins, with a high degree of population differences in disease prevalence or outcomes. These factors include, but are not limited to, epigenetic factors regulating the expression of drug-metabolizing enzymes and transport proteins [20]; non-genetic covariate factors such as age, gender, race and height [21,22]; interindividual variability in the gut microbiome, influencing metabolism and bioavailability [23]; pathophysiological conditions such as diminished kidney and liver function [24]; other factors such as polypharmacy resulting in pharmacokinetic drug–drug interaction [25,26,27]; environmental factors, such as smoking, alcohol intake and medication causing CYP450 enzyme induction or inhibition, resulting in an altered phenotype [5,28]; short-term fasting [29]; and certain foods and herbal remedies that may also influence the phenotypic expression of specific CYP450 enzymes [30,31,32].

When considering a more individualized approach to pharmacotherapy, it is clear that genotyping alone cannot infer altered metabolic or transport phenotypes, considering the complex interaction between genotype and extrinsic factors influencing metabolic or transport activity [5]. Genotype–phenotype mismatches due to the co-administration of medications or comorbidities, altering the clinical metabolizer phenotype, have been reported in a number of studies [5,33,34,35]. This phenomenon is called phenoconversion and describes a situation where phenotypic responses contradict the measured genotype [36]. Hiemke et al. noted that phenotyping may provide an advantageous alternative where the functional significance of genetic polymorphisms are unclear [37], providing a real-time snapshot of individual metabolism or transport activity that take all influencing factors into account.

The aim of this study was therefore to critically review phenotyping cocktails aimed at assessing the real-time in vivo CYP450 metabolic activity and P-gp activity for feasibility of use in routine clinical practice within a Sub-Saharan African context and to identify challenges in the implementation thereof.

## 2. Review of Phenotyping Cocktails Developed over the Last Two Decades

A literature search conducted using the University of Pretoria’s library databases (36 databases for Health Sciences, including AccessMedicine, BMJ, ClinicalKey, MEDLINE (Ovid), PubMed, Scopus and TOXLINE) was limited to in vivo cocktails used in human populations consisting of five or more probe drugs to phenotype phase I metabolizing enzymes and/or the P-gp transporter with a cocktail approach. Only articles available in the English language were included. A summary of the multiple probe phenotyping cocktails is given in Table 1, listing the sampling matrix, the enzyme and/or transporter investigated in the cocktail with the corresponding phenotyping drug and dosage, the phenotyping metric (i.e., concentration–time profiles with drug area under the curve (AUC), probe-drug-to-metabolite concentration ratio in plasma/urine or absolute urinary recovery) used to assess metabolic or transport activity and bioanalytical methods used for quantitation.

At present, phenotyping cocktails, containing multiple probe drugs, are used for the simultaneous assessment of drug metabolism during drug development in drug–drug interaction and toxicology studies and regulated by the EMA and the FDA [38]. Due to the safety concerns of possible drug–drug interactions with new chemical entities (NCE), this has to be clinically evaluated during early drug development. Earlier cocktails used plasma and urine sampling to phenotype mostly phase I metabolism [39,40,41,42,43,44,45,46] and in some cocktails also phase II metabolism [47,48,49]. Three of the recent multiple drug cocktails included a P-gp probe, either digoxin [50] to assess renal P g activity or fexofenadine [51,52] assessing intestinal P-gp transport. Alternative non-invasive sampling strategies, using DBS and/or saliva, were explored in two cocktails, namely the Geneva [51] and Basel [53] cocktails.

During the validation of phenotyping cocktails, pilot PK studies were conducted as a proof of concept for use in human populations, and most of the reviewed cocktails included healthy non-smoking male subjects [15,19,23,26,28,37] or healthy male and female cohorts [17,22,25,27,38], with the sample sizes varying from three to thirty-three. Two groups tested their phenotyping cocktails on patient cohorts: Ghassabian et al. [31] assessed 11 patients with schizophrenia, and Grangeon et al. [39] simultaneously assessed the systemic and urinary clearance of a new drug using 30 patients on polypharmacy during a clinical trial.

Although some cocktail studies included genotyping, the objective was not to infer genotype–phenotype relationships, but rather to exclude certain genotypes or as an exploratory analysis of interindividual variation. For the Pittsburg 2006 cocktail [24] for example, two of the volunteers were homozygous for the CYP2D6*4 allele, and by removing their phenotypic data from the analysis, the intersubject CV % decreased from 44.8 to 31.9%.

**Table 1:** Summary of in vivo phenotyping cocktails with five or more probes used in human populations during the past 20 years.

Cocktail (n)	Matrix	PKP	Probe Drugs and Doses	Phenotyping Metrics	Analytical Methods	Ref.
1999 “GW cocktail” (n = not specified)	Plasma and Urine	CYP1A2 CYP2C9 CYP2C19 CYP2D6 CYP2E1 CYP3A4	caffeine 100 mg diclofenac 10 mg mephenytoin 25 mg debrisoquine 10 mg chloroxazone 250 mg midazolam 5 mg	Concentration–time profiles for caffeine, chloroxazone, midazolam and metabolites. Absolute urinary recovery over 12 h for *S*-mephenytoin and diclofenac.	Online-SPE LC-MS/MS	[39]
2001 Zhu et al. (n = 14)	Plasma and Urine	CYP1A2 CYP2C19 CYP2D6 CYP2E1 CYP3A4	caffeine 100 mg mephenytoin 100 mg metoprolol 100 mg chloroxazone 200 mg midazolam 7.5 mg	[par]/[caf] _6 h plasma_ [mep]/[OH-mep] _8 h collective urine_ [met]/[OH-met] _8 h collective urine_ [OH-chlor]/[chlor] _4 h plasma_ [OH-mdz]/[mdz] _1 h plasma_	β-glucuronidation + liquid extraction LLE HPLC-UV	[40]
2003 Karolinska cocktail (n = 24)	Plasma and Urine	CYP1A2 CYP2C9 CYP2C19 CYP2D6 CYP3A4	caffeine 100 mg losartan 25 mg omeprazole 20 mg debrisoquine 10 mg quinine 250 mg	[par]/[caf] _3.5, 4 h plasma_ [los]/[E 3174] _8 h collective urine_ [OH-opz]/[opz] _3, 3.5 h plasma_ [deb]/[OH-deb] _8 h collective urine_ [OH-qui] _16 h plasma_	PPT of plasma with ACN, LLE HPLC-UV HPLC-FL detection	[41]
2003 Cooperstown 5 + 1 cocktail (n = 12)	Plasma and Urine	CYP1A2, NAT2, XO CYP2C19 CYP2D6 CYP3A4	caffeine 2 mg/kg caffeine 2 mg/kg caffeine 2 mg/kg omeprazole 40 mg dextromethorphan 30 mg midazolam 0.025 mg/kg (plus, vit K) S-warfarin 10 mg	[1X + 1U + AFMU]/[17U]_12 h collective urine_ [AFMU]/[1X + 1U] _12 h collective urine_ [1U]/[1X + 1U] _12 h collective urine_ [OH-opz]/[opz] _plasma_ [dtp]/[dex] _12 h collective urine_ [OH-mdz]/[mdz] _plasma_ AUC _0–∞ *S-*warfarin_	LLE, SPE HPLC-UV HPLC-FL detection	[47]
2004 Quebec cocktail Sharma et al. (n = 10)	Urine	CYP1A2, NAT2, XO CYP2C9 CYP2D6 CYP2E1 CYP3A4	caffeine 100 mg caffeine 100 mg caffeine 100 mg tolbutamide 250 mg metoprolol 25 mg chloroxazone 250 mg dapsone 100 mg	[1X + 1U + AFMU]/[17U]_8 h collective urine_ [AFMU]/[AFMU + 1X + 1U] _8 h collective urine_ [1U]/[1X + 1U] _8 h collective urine_ [COOH-tol + OH-tol]/[tol] _8 h collective urine_ [Met]/[OH-met] _8 h collective urine_ [OH-chlor]/[chlor] _8 h collective urine_ [dap-HA]/[dap + dap-HA] _8 h collective urine_	β-glucuronidase/arylsulphatase + LLE HPLC-UV LC-MS/MS	[48]
2004 Loughborough -Blakey et al. (n = 12)	Plasma and Urine	CYP1A2 CYP2C9 CYP2D6 CYP2E1 CYP3A4	caffeine 100 mg tolbutamide 250 mg debrisoquine 5 mg chloroxazone 250 mg midazolam 0.025 mg/kg	[par]/[caf] _6.5 h plasma_ [COOH-tol + OH-tol]/[tol] _6–12 h urine_ [deb]/[OH-deb] _0–6 h urine_ [OH-chlor]/[[chlor] _2 h 32 min plasma_ AUC _last plasma MDZ_	Dilute and shoot/β-glucuronidase +/SPE/ACN PPT LC-MS	[42]
2004 Jerdi et al. (Geneva University Hospital) (n = 10)	Plasma	CYP1A2 CYP2C9 CYP2C19 CYP2D6 CYP3A4	caffeine 100 mg flurbiprofen 50 mg omeprazole 40 mg dextromethorphan 25 mg midazolam 7.5 mg	PK parameters and clinical study were to be published elsewhere. No reference found in English language.	LLE/PPT HPLC-UV and HPLC-FL detection	[54]
2004 Yin et al. (n = 16)	Plasma and Urine	CYP1A2 CYP2C9 CYP2C19 CYP2D6 CYP3A4	caffeine 100 mg tolbutamide 500 mg omeprazole 40 mg debrisoquine 10 mg midazolam 3.75 mg	[par]/[caf] _2/3 h plasma_ [COOH-tol + OH-tol]/[tol] _6–12 h urine_ [OH-opz]/[opz] _2/3 h plasma_ [OH-deb]/[deb] _0–6 h urine_ [OH-mdz]/[mdz] _2/3 h_	SPE LC-MS	[43]
2005 Tomalik-Scharte et al. (Note: 30 mg of dextromethorphan-HBr also given, results not reported) (n = 16)	Plasma and Urine	CYP1A2 CYP2C9 CYP2C19 CYP3A4 _Hepatic_ CYP3A4 _Intestinal_	caffeine 150 mg tolbutamide 125 mg mephenytoin 50 mg midazolam 2 mg iv midazolam 1 mg po	[par]/[caf] _6 h plasma_ [COOH-tol + OH-tol]/[tol] _6–12 h urine_ AND AUC_0–∞,_ C_max oral_, t_max oral_, t _1/2, λz_, CL/F, [tol] _24 h plasma_ 4′-Hydroxymephenytoin _0–8 h urine_ AUC _0–∞ i.v._, CL _i.v. mid_, F_hepatic_ F_oral_, F_intestinal_, AUC_0--∞ oral_, C_max oral_, t_max oral_, t _1/2, λz_	β-glucuronidase deconjugation/SPE/plasma PPT HPLC-UV LC-MS/MS	[44]
2006 Pittsburg + 1 (n = 24)	Plasma and Urine	CYP1A2 CYP2C9 CYP2C19 CYP2D6 CYP2E1 NAT2	caffeine 100 mg flurbiprofen 50 mg mephenytoin 100 mg debrisoquine 10 mg chloroxazone 250 mg dapsone 100 mg	[par]/[caf] _8 h plasma_ [OH-flb]/[OH-flb + flb] _0–8 h urine_ 4′-Hydroxymephenytoin _0–8 h urine_ [OH-deb]/[OH-deb + deb] _0–8 h urine_ [OH-chlor]/[chlor] _4 h plasma_ [MA-dap]/[dap] _8 h plasma_	No sample prep mentioned HPLC	[49]
2006 Darmstadt-Krösser et al. (n = 18)	Plasma and Urine	CYP1A2 CYP2C9 CYP2C19 CYP2D6 CYP3A4	caffeine 100 mg diclofenac 50 mg mephenytoin 100 mg metoprolol 100 mg midazolam 7.5 mg	AUC _0–24 h par_/AUC _0–24 h caf_ AUC _0–24 h OH-dic_/AUC _0–24 h dic_ 4′-Hydroxymephenytoin _0–8 h urine_ AUC _0–72 h OH-met_/AUC _0–72 h met_ AUC_0–24 mdz_	SPE HPLC-FL LC-MS/MS	[46]
2007 Inje cocktail (n = 12)	Plasma and Urine	CYP1A2 CYP2C9 CYP2C19 CYP2D6 CYP3A4	caffeine 93 mg losartan 30 mg omeprazole 20 mg dextromethorphan 30 mg midazolam 2 mg	[par]/[caf] _4 h plasma_ [los]/[E 3174] _8 h collective urine_ [OH-opz]/[opz] _4 h plasma_ log[dtp]/[dex] _8 h collective urine_ [mdz] _4 h plasma_	LLE LC-MS/MS HPLC-FL detection	[45]
2008 Petsalo et al. (n = not specified)	Urine	CYP1A2 CYP2A6 CYP2B6 CYP2C8 CYP2C9 CYP2C19 CYP2D6 CYP2E1 CYP3A4 CYP3A4	melatonin 3 mg nicotine 2 mg bupropion 150 mg repaglinide 1 mg losartan 50 mg omeprazole 20 mg dextromethorphan 12.5 mg chloroxazone 62.5 mg midazolam 3.75 mg omeprazole 20 mg	[mel] AND [OH-mel] _8 h collective urine_ [nic] AND [cot] _8 h collective urine_ [bup] AND [OH-bup] _8 h collective urine_ [rep] AND [OH-rep] _8 h collective urine_ [los] AND [E 3174] _8 h collective urine_ [opz] AND [OH-opz] _8 h collective urine_ [dex] AND [dtp] _8 h collective urine_ [chlor] AND [OH-chlor] _8 h collective urine_ [mdz] AND [OH-mdz] _8 h collective urine_ [opz] AND [opz-sulphone] _8 h collective urine_	β-glucuronidase hydrolysis UPLC-MS/MS LC-MS/MS	[55]
2009 Ghassabian et al. (n = 11)	Plasma	CYP1A2 CYP2C9 CYP2C19 CYP2D6 CYP3A4	caffeine 100 mg losartan 25 mg omeprazole 20 mg dextromethorphan 30 mg midazolam 2 mg	[par]/[caf] _4 h_ AUC _0–6 h E-3174_/AUC _0–6 h los_ [OH-opz]/[opz] _4 or 6 h_ AUC _0–6 h dtp_/AUC _0–6 h dex_ AUC _0–6 h OH-mdz_/AUC _0–6 h mdz_	SPE and LLE after initial PPT with CAN HPLC-MS/MS	[56]
2009 Sanofi-Aventis cocktail-Turpault et al. (n = 30)	Plasma	CYP1A2 CYP2C9 CYP2C19 CYP2D6 CYP3A4	caffeine 100 mg *S*-warfarin 10 mg omeprazole 20 mg metoprolol 100 mg midazolam 0.03 mg/kg _IV_	AUC_0–∞ caffeine_ AUC_0–∞ *S-*warfarin_ AUC_0–∞ omeprazole_ AUC_0–∞ metoprolol_ AUC_0–∞ midazolam_	SPE and LLE LC-MS/MS separate analysis	[57]
2010 CIME cocktail NOTE: initial cocktail included amodiaquine as CYP2C8 probe. Repaglinide was added in 2016 (n = not specified)	Plasma	CYP1A2 CYP2C8 CYP2C9 CYP2C19 CYP2D6 CYP3A4 OATP UGT Renal P-gp	caffeine 73 mg repaglinide 0.25 mg * tolbutamide 10 mg omeprazole 10 mg dextromethorphan 18 mg midazolam 4 mg rosuvastatin 5 mg acetaminophen 60 mg memantine 5 mg digoxin 0.25 mg	C_max_, AUC_∞_, t_1/2_, CL/F were calculated for all substrates in addition to AUC_∞substrate_/AUC_∞metabolite_ for CYP450 substrates and metabolites.	SPE UPLC-MS/MS	[50,58]
2012 Inje–low dose Oh et al. (n = 13)	Plasma	CYP1A2 CYP2C9 CYP2C19 CYP2D6 CYP3A4	caffeine 10 mg losartan 2 mg omeprazole 200 µg dextromethorphan 2 mg midazolam 100 µg	AUC_0–12 h caf_, AUC _0–12 h par_ AUC_0–12 h los_, AUC _0–12 h EXP3174_ [OH-opz] _1.5 h_, [opz] _1.5 h_ AUC_0–12 h dex_, AUC _0–12 h dtp_ C_max OH-mdz at 6 h_, AUC _0–12 h OH-mdz_	LLE LC-MS/MS	[59]
2012 Wohlfarth et al. (n = 14)	Plasma	CYP1A2 CYP2C9 CYP2C19 CYP2D6 CYP3A4	caffeine 100 mg tolbutamide 125 mg omeprazole 20 mg dextromethorphan 30 mg midazolam 2 mg	[par]/[caf] _4 h_ [tol] _24 h plasma_ [OH-opz]/[opz] _4 h_ [dex]/[dtp] _4 h_ [mdz] _4 h_	SPE LC-MS/MS	[60]
2014 Geneva cocktail (n = 10)	Plasma and DBS	CYP1A2 CYP2B6 CYP2C9 CYP2C19 CYP2D6 CYP3A4 P-gp	caffeine 50 mg bupropion 20 mg flurbiprofen 10 mg omeprazole 10 mg dextromethorphan 10 mg midazolam 1 mg fexofenadine 25 mg	[par]/[caf] _2 h_ [OH-bup]/[bup] _3 h_ [OH-flb]/[flb] _3 h_ AUC_2,3,6 h opz_/AUC_2,3,6 h OH-opz_ [dtp]/[dex] _3 h_ [OH-mdz]/[mdz] _2 h_ Limited sampling AUC_2,3,6 h_	DBS—MeOH Plasma—ACN PPT LC-MS/MS	[51,61]
2014 Basel cocktail (n = 16)	Plasma, saliva and DBS	CYP1A2 CYP2B6 CYP2C9 CYP2C19 CYP2D6 CYP3A4	caffeine 100 mg efavirenz 50 mg losartan 12.5 mg omeprazole 10 mg metoprolol 12.5 mg midazolam 2 mg	[par]/[caf] _8 h plasma;_ [par]/[caf] _8 h DBS;_ [par]/[caf] _8 h saliva_ [efv]/[OH-efv] _8 h plasma_ [los]/[E 3174] _8 h plasma_ [opz]/[OH-opz] _2h plasma; _ [opz]/[OH-opz] _2 h DBS; _ [opz]/[OH-opz] _2 h saliva_ [met]/[OH-met] _8 h plasma_ [mdz]/[OH-mdz] _2 h plasma_	PPT LC-MS/MS	[53]
2016 Lammers et al. (n = not specified)	Plasma	CYP1A2 CYP2C9 CYP2C19 CYP2D6 CYP3A4	caffeine 100 mg warfarin 5 mg omeprazole 20 mg metoprolol 100 mg midazolam 0.03 mg/kg _IV_	AUC_0–∞ caffeine_ AUC_0–∞ *S-*warfarin_ AUC_0–∞ omeprazole_ AUC_0–∞ metoprolol_ AUC_0–∞ midazolam_	PPT with 42:8 ACN: MeOH LC-MS/MS nonchiral and chiral methods	[62]
2017 Puris et al. NOTE: repaglinide excluded as metabolite 3′-hydroxyrepaglinide not detected from samples and interference of another compound with similar *m*/*z* (n = 4)	Urine and Serum	CYP1A2 CYP2A6 CYP2B6 CYP2C8 CYP2C9 CYP2C19 CYP2D6 CYP2E1 CYP3A4 CYP3A4	melatonin 2 mg nicotine 1 mg bupropion 37.5 mg repaglinide 0.25 mg losartan 12.5 mg omeprazole 10 mg dextromethorphan 30 mg chloroxazone 62.5 mg midazolam 1.85 mg omeprazole 10 mg	AUC_0–6 h limited sampling_, C_max_ and t_max_ and cumulative concentration in urine for probe drugs and metabolites calculated. 5-Hydroxyomeprazole indicative of CYP2C19 metabolism and omeprazole sulfone of CYP3A4 metabolism.	β-glucuronidase hydrolysis for urine SPE, PPT (method of choice), LLE LC-MS/MS—3 separate runs	[63]
2017 Grangeon et al. NOTE: chlorzoxazone administered separately to avoid interaction with CYP3A4 (n = not specified)	Plasma and Urine	CYP1A2 CYP2B6 CYP2C9 CYP2C19 CYP2D6 CYP3A4 CYP2E1	caffeine 100 mg bupropion 100 mg tolbutamide 250 mg omeprazole 20 mg dextromethorphan 30 mg midazolam 2 mg chlorzoxazone 250 mg	Plasma and urinary concentrations of all probe drugs and metabolites were obtained from patients on polypharmacy.	β-glucuronidase/sulfatase hydrolysis PPT Three separate UPLC-MS/MS methods	[64]
2018 Sao Paulo cocktail (n = 3)	Plasma	CYP1A2 CYP2C9 CYP2C19 CYP2D6 CYP3A4 P-gp	caffeine 10 mg losartan 2 mg omeprazole 2 mg metoprolol 10 mg midazolam 0.2 mg fexofenadine 10 mg	AUC_0–∞_ for all analytes except E-3174 where AUC_0–12 h_ were used, C_max_ and Cl/F (L/h).	SPE, LLE, PPT Three separate UPLC-MS/MS methods	[52]

(n)—number of subjects phenotyped in the validation of the cocktail; PKP—pharmacokinetic parameters; AUC—area under the plasma concentration time curve; UR—urinary recovery ratio; MR—metabolic ratio [parent]/[metabolite]; CYP—cytochrome P450 enzyme; NAT2—N-acetyltransferase 2; XO—xanthine oxidase; OATP—organic-anion-transporting polypeptide; UGT—uridine diphosphate glycosyltransferase; P-gp—permeability glycoprotein; par—paraxanthine; caf—caffeine; mep—*S*-mephenytoin; OH-mep—4′-hydroxymephenytoin; met—metoprolol; OH-met—α-hydroxymetoprolol; OH-chlor—6′-hydroxychloroxazone; chlor—chlorzoxazone; OH-mdz—1′-hydroxymidazolam; mdz—midazolam; los—losartan; E 3174—active losartan metabolite; OH-opz—5′-hydroxy-omeprazole; opz—omeprazole; deb—debrisoquin; OH-deb—4′-hydroxydebrisoquine; OH-qui—3′-hydroxyquinine; 1X—1-methylxanthine; 1U—1-methylurate; AFMU—5-acetylamino-6-formylamino-3-methyluracil; 17U—1,7-dimethylurate; dtp—dextrorphan; dex—dextromethorphan; COOH-tol + OH-tol—carboxytolbutamide + methylhydroxytolbutamide; tol—tolbutamide; dap-HA—dapsone hydroxylamine; dap—dapsone; OH-flb—hydroxyflurbiprofen; flb—flurbiprofen; MA-dap—dapsone; OH-dic—hydroxydiclofenac; dic—diclofenac; mel—melatonin; OH-mel—hydroxymelatonin; nic—nicotine; cot—cotinine; rep—repaglinide; OH-rep—hydroxurepaglinide; efv—efavirenz; OH-efv—hydroxy-efavirenz; OH-bup—hydroxy-bupropion; bup—bupropion; C_max_—maximum plasma concentration; t_max_,—time to reach maximum plasma concentration; t_1/2 λz_—terminal half-life; F_intestinal_—intestinal availability of midazolam; changes in intestinal CYP3A4 activity were calculated as the inverse of changes in F_intestinal_; SPE—solid-phase extraction; LLE—liquid–liquid extraction; PPT—protein precipitation; MeOH—methanol; ACN—acetonitrile; HPLC-MS/MS—high-performance liquid chromatography tandem mass spectrometry; HPLC-UV—high-performance liquid chromatography ultraviolet detection; HPLC-FL—fluorescence detection; DBS—dried blood spots on Whatman filter paper 903.

## 3. Discussion

Although most of the 24 phenotyping cocktails in Table 1 are fit for purpose when it comes to drug development and DDI studies of NCEs, their limitations of use in clinical phenotyping towards individualized therapy can be summarized as follows (Table 2).

Despite the use of drug cocktails during drug development, routine phenotyping in clinical practice towards individualized pharmacotherapy has not yet become reality. The only example of routine phenotyping in clinical practice is the determination of phenylalanine in small volumes of blood (DBS) or urine in newborn infants, for phenylketonuria screening [65]. For clinical applicability, phenotyping cocktails are scrutinized for their ability to use probe drugs that are widely available with acceptable safety profiles, selective to specific CYP enzymes or P-gp and other transporters and well tolerated at the doses given to patients, with an uncomplicated route of administration and sampling procedures. Herein, a single matrix assay would promote the implementation of phenotyping in routine practice, especially when coupled with limited sampling procedures. Non-invasive sampling would be advantageous to obtain an estimation of metabolic or transport activity at baseline or to continuously assess the causes of unexpected drug plasma concentration during treatment. Urine sampling, proposed in many cocktails, is non-invasive but confounded by sampling errors, urinary pH and glomerular filtration rate, attributing to the high intraindividual variability found in dextromethorphan [66] and caffeine [67] urinary metabolic ratios. Metabolite to parent single time point ratios in urine also proved to be problematic in clinical trials where extrapolation into sound dosing guidelines is a necessity. Phenotyping cocktails should also exhibit minimal PK or PD interaction (i.e., interference in absorption, metabolism or clearance or at the receptor site). The analytical interaction between multiple drugs administered together should be evaluated during sample preparation, detection and quantitation [56]. Fuhr et al. made reference to the fact that the chosen probe drugs and the phenotype identifying measurement, derived from assessing quantitative change in the biological response to the probe drug, must further provide an accurate estimate of the real-time in vivo biological activity, must be applicable to other substrates used to phenotype the same enzyme or transporter and should reflect changes in their biological activity in the presence of inhibitors or inducers [68].

### 3.1. Selectivity of Probe Drugs for Metabolizing Enzymes or Drug Transporters

The first main problem of current probes suggested by the FDA for phenotyping is the fact that no probe drug is completely selective for a single metabolizing enzyme or transporter. Nonetheless, the contribution of a specific pharmacokinetic pathway to the disposition of the probe drug should be primary and in addition must be indicative of changes in the phenotype when subject to an inducer or inhibitor [38]. For example, caffeine, a fully validated probe for CYP1A2, is also metabolized by CYP2E1, N-acetyl-transferase 2 (NAT2) and xanthine oxidase (XO) enzymes, but since CYP1A2 is the dominant metabolic pathway [69], most cocktails use the metabolic ratio of paraxanthine to caffeine plasma concentration [41,45,49,51,53,56] as a CYP1A2 phenotype identifier. Alternatively, provided the phenotyping measurement is carefully chosen, all metabolites of caffeine could be quantified to assess NAT2 and XO activity simultaneously, as in the Cooperstown [47] and Quebec [48] cocktails. Similarly, the metabolism of omeprazole to its hydroxylated metabolite and sulfone metabolite has been used to simultaneously assess CYP2C19 and CYP3A4 metabolism, respectively, in a recent cocktail [63]. Tolbutamide is an almost exclusive probe for CYP2C9, but the proposed phenotyping measurement of 24 h plasma concentration would restrict its usefulness in routine phenotyping. Metoprolol has been studied as a selective probe for CYP2D6 metabolism, but correlation with other CYP2D6 probes could not be established in an African population from Tanzania carrying a population-specific CYP2D6*17 allele [70], raising questions about its usefulness as a probe. This discordance between genotype and observed phenotype with altered substrate specificity in African populations has been shown in a number of studies [71,72,73]. These findings confirm the need for further research on different population groups before routine phenotyping can be implemented in clinical practice.

Phenotyping drug transporter activity may also provide a useful metric to assess and predict drug absorption or excretion (depending on the location of the drug transporter protein) in vivo [68]. The role of transporters in drug–drug interactions and the clinical safety and efficacy of drugs has been the focus of the International Transport Consortium since 2010 [6]. In a review by Ma et al., evaluating four P-gp probes, none met all the proposed validation criteria for an ideal probe drug [74]. Both digoxin and fexofenadine have overlapping substrate specificities with other transporters and their correlation with other P-gp probes was not established; in addition, digoxin has a narrow therapeutic window, limiting its usefulness as a probe in patient populations. Despite the fact that no ideal P-gp probe exist, fexofenadine is safe and has been used in phenotyping drug cocktail studies [51,52] and pharmacokinetic studies [75,76,77]. Understanding the pharmacokinetic processes influenced by xenobiotic exposure, the site of exposure and the expression and distribution of metabolizing enzymes and transporters at that site is imperative for assigning phenotype and making clinical decisions based on that assessment.

The chosen probe drugs should clearly elucidate the in vivo pharmacokinetic phenotype under investigation, and overlapping substrate specificities between P-gp and CYP3A4 in particular should be considered. A higher expression of CYP3A4 in enterocytes will significantly influence the first pass bioavailability of CYP3A4 substrates and therefore if the objective is to phenotype hepatic CYP3A4 activity, probe substrates should be administered by the intravenous route [78]. Changes in substrate selectivity for metabolizing enzymes and transporters when administered at lower subtherapeutic doses must be considered with the validation of low-dose cocktails. In most cases, a lower substrate dose will increase drug selectivity; however, even validated cocktails have to be re-evaluated when the dosages are lowered to ensure the applicability of the phenotype assessments [78]. An important factor to consider is dose-dependent plasma protein binding, as a result of the saturation of the available binding sites, influencing the fraction of unbound drug in systemic circulation as explained by Macheras and Rosen [79]. Micro dose strategies with phenotyping cocktails, containing dosages 100-fold lower than the normal dosages, have been proposed, but the authors stress that linear pharmacokinetics between normal and micro doses are required for the correct prediction of enzyme or transport activity. This is due to the fact that protein binding may be dose-dependent and both decreased bioavailability or the non-saturation of compartments during drug distribution may lead to non-linear pharmacokinetics. Furthermore, very precise and sensitive quantitation methods are required [80].

### 3.2. Tolerability of Drug Doses Used in Phenotyping Cocktails and Safety Profiles of Some Proposed Probes

Secondly, earlier cocktails contained probe drugs at therapeutic doses, contributing to possible side effects, especially considering drugs with narrow therapeutic indexes, such as tolbutamide, warfarin and digoxin. Any small variation in enzyme or transport activity could contribute greatly to the disposition of drugs with a narrow therapeutic index, causing severe adverse reactions. Possible side effects with therapeutic probe drug doses included hypotension with debrisoquin (CYP2D6 probe), hypoglycemia with tolbutamide [81] (CYP2C9 probe), bleeding risk with warfarin (CYP2C9 probe, requiring co-administration of vitamin K) and gastrointestinal side effects and sedation with mephenytoin (CYP2C19) [82]. The incidence of side effects has been largely eliminated since the introduction of low-dose phenotyping cocktails; however, they present pharmaceutical complications, because probe drugs are not commercially available at these low doses and have to be compounded from available dosage forms. More importantly, low-dose phenotyping cocktails require optimized, sensitive bioanalytical methods to detect low concentrations of metabolites in biological matrixes, especially when probe drugs and their metabolites, all with different physicochemical properties, are to be simultaneously quantified in a single run. An example of an ideal probe drug is flurbiprofen for phenotyping CYP2C9. It is almost exclusively metabolized by this enzyme, has a wide therapeutic window and is not dependent on urinary conjugation for excretion; therefore, it has a much better safety profile than tolbutamide and warfarin [83], justifying its incorporation into the Pittsburg cocktail [49].

### 3.3. Sample Collection Protocols and Corresponding Phenotyping Measurements Chosen for Phenotype Assessment

A third main challenge of current proposed phenotyping cocktails is the inconvenient and impractical sample collection protocols. Multiple time point venous plasma sampling or collective urine sampling would not be feasible in a routine clinical environment. Use of a single or limited time point sampling strategy to measure metabolic or transporter activity would be advantageous especially when coupled with probe drugs with short elimination half-lives to reduce the time patients have to spend at the clinic for observation. Studies comparing the systemic clearance (AUC) of probe drugs or the clearance ratio of probe drug to metabolite to limited AUC or single time point metabolic ratios are currently underway [53,84,85,86,87,88]. No consensus has yet been reached and results are conflicting. In validating their Basel phenotyping cocktail, Donzelli et al. correlated the AUC_0–24 h_ ratios for probe versus metabolite to a number of single time point plasma metabolic ratios (see Table 1) including a 2 h single time point midazolam metabolic ratio (r^2^ of 0.959). Yang et al., on the other hand, found a 4 h limited sampling AUC for midazolam and a 4 h single time point concentration to best fit a two-compartmental population PK model, derived from 2122 observations from 152 healthy subjects, for the estimation of CYP3A4 metabolic activity [87]. A 5 h single time point plasma midazolam concentration [89] and limited sampling at 0.5, 2 and 6 h for midazolam [84] have also been suggested. Similarly, many single time point paraxanthine over caffeine metabolic ratios have been shown to correlate with the systemic clearance of caffeine, ranging from 2 h [51], 4 h [56] and 8 h [53] post oral dose. Care should be taken in choosing the phenotyping measurement to infer metabolic or transport activity in different patient populations. Chosen phenotyping measures should be validated; correlate with enzyme or transport activity and represent change clearly under induction or inhibition conditions; account for confounding factors such as glomerular filtration rate or urinary pH; and have low intra-individual variability [69,78]. Intraindividual variability is usually lower with plasma sampling rather than urinary sampling.

### 3.4. Pharmacokinetic, Pharmacodynamic and Bioanalytical Interaction between Probe Drugs in Simultaneous Assessment of Phenotype

An understanding of the PK and PD interaction between probe drugs used together in a cocktail approach is essential. Interactions at the target receptor sites (PD interactions) should also be considered; for example, using the antihypertensives losartan and debrisoquin together might cause hypotension. Each probe drug used in a proposed cocktail must be validated individually and then in combination to exclude interaction with other probe drugs. In the Basel cocktail, chlorzoxazone (a CYP2E1 probe) had to be excluded due to a significant interaction with CYP3A4, significantly increasing midazolam AUC_0–24h_ when administered together [53]. To overcome this, Blakey et al. administered the midazolam intravenously to exclude this intestinal CYP3A4 interaction with chlorzoxazone [42]. Although separate intravenous dosing is feasible during drug interaction studies and during drug development, it would be difficult to implement in clinical practice. Chlorzoxazone also interacts with CYP1A2 and when administered together with caffeine caused a 16–20% decrease in caffeine metabolism in urine and plasma [90]. Simultaneous probe drug and metabolite quantitation using bioanalytical methods requires optimization due to different physicochemical properties to reduce competition for charge and to optimize individual extraction recovery, ionization efficiency and detection limits.

## 4. Conclusions and Future Direction

Pharmacokinetic variability is caused by a complex interplay between many different factors influencing the available drug concentration in the body. Measuring specific drug concentrations of substrates for either metabolizing enzymes or drug transporter proteins provides a fingerprint of metabolic or transport activity in vivo, which is then correlated with the real-time phenotype. Unlike the functional genotype, which depends on epigenetic regulation or post-translational modifications, this approach measures biochemical activity directly correlated with functional phenotype. It considers all intrinsic and extrinsic factors influencing variability in a dynamic way, because this will change depending on pathophysiology, age, lifestyle and co-medication changing over time for an individual, and should therefore be carried out routinely in order to assist clinicians in drug selection and dosing toward personalized pharmacotherapy. This, in turn, could help to reduce the incidence of ADR, DDI and therapeutic failure seen in Africa.

A number of phenotyping cocktails aimed at assessing in vivo CYP450 metabolic activity and in some instances P-gp activity have been developed, but their implementation in clinical practice has been limited by a wide variety of challenges, as set out above. Non-invasive sampling could be advantageous for implementing phenotyping in routine practice to obtain an estimation of metabolic or transport activity at baseline or for therapeutic drug management, especially in genetically diverse population groups. In this regard, dried blood spot (DBS) sampling can be used to simultaneously assess P-gp and CYP activity with a low-dose phenotyping cocktail and limited sampling to measure pharmacokinetic markers and, by extension, to measure phenotype. Before DBS sampling can be implemented in routine clinical practice, the question remains as to whether the quantitative bioanalysis of drugs in a capillary whole-blood matrix correlates with that of the standard plasma/serum matrixes used as a reference in the current clinical environment. When using alternative sampling strategies to the gold-standard plasma sampling, it is important that future studies assess the distribution of the expressed enzymes or transporters under investigation and the pharmacokinetic processes involved, i.e., absorption or excretion rates and drug distribution in different physiological compartments.

## Figures and Tables

**Table 2:** Limitations of in vivo phenotyping cocktails for application in routine clinical practice.

Limitation	Number of Cocktails with the Limitation	References
Multiple routes of administration	5	[42,44,47,57,62]
Use of both urine and plasma matrixes in the phenotype assessment	14	[39,40,41,42,43,44,45,46,47,49,63,64]
Discontinuation of probes mephenytoin and debrisoquin in most countries	8	[39,40,41,42,43,44,46,49]
Use of therapeutic doses eliciting side effects in earlier cocktails	10	[39,40,41,42,43,46,47,48,49,54]
Interaction between probe substrates requiring separate administration time points	7	[39,40,41,42,55,63,64]
Extensive sampling procedures	15	[39,40,41,42,43,44,45,46,47,48,49,52,53,55,59]
Complicated sample workup or multiple extraction assays	8	[41,42,44,48,52,54,56,57]
Impractical analytical procedures		
Multiple bioanalytical methods used in a single cocktail	5	[52,57,62,63,64]
Outdated analytical instruments with low detection limits	4	[40,41,47,54]

## Data Availability

No new data were created or analyzed in this study. Data sharing is not applicable to this article.
